# Factors that determine energy compensation: a systematic review of preload studies

**DOI:** 10.1111/nure.12048

**Published:** 2013-06-10

**Authors:** Eva Almiron-Roig, Luigi Palla, Kathryn Guest, Cassandra Ricchiuti, Neil Vint, Susan A Jebb, Adam Drewnowski

**Affiliations:** Elsie Widdowson Laboratory, MRC Human Nutrition ResearchCambridge, UK; Department of Clinical Sciences, University of ChesterChester, UK; School of Public Health, University of WashingtonSeattle, Washington, USA

**Keywords:** intermeal interval, physical form, satiety, weight management

## Abstract

Insufficient energy compensation after a preload (meal, snack, or beverage) has been associated with excess energy intake, but experimental studies have used heterogeneous methodologies, making energy compensation difficult to predict. The aim of this systematic review was to analyze the relative contributions of two key variables, preload physical form and intermeal interval (IMI), to differences in energy compensation. Forty-eight publications were included, from which percent energy compensation (%EC) data were extracted for 253 interventions (121 liquid, 69 semisolid, 20 solid, and 43 composite preloads). Energy compensation ranged from −370% (overconsumption, mostly of liquids) to 450% (overcompensation). A meta-regression analysis of studies reporting positive energy compensation showed that IMI (as the predominant factor) together with preload physical form and energy contributed significantly to %EC differences, accounting for 50% of the variance, independently from gender and BMI. Energy compensation was maximized when the preload was in semisolid/solid form and the IMI was 30–120 min. These results may assist in the interpretation of studies assessing the relative efficacy of interventions to enhance satiety, including functional foods and weight management products.

## Introduction

Energy compensation is defined as the adjustment of energy intake provoked by the previous ingestion of a given stimulus (preload), whether a meal, a snack, or a beverage.^1^ Insufficient energy compensation both in the short and the long term has been associated with increased energy intakes and positive energy balance, leading to obesity.^2^,^3^ Understanding the various influences on energy compensation may assist in controlling energy intake and inform obesity prevention strategies.

Studies focusing on the short-term regulation of energy intake typically employ a preload paradigm, where the effects of a food attribute on subsequent eating can be evaluated.^4^ This design allows direct observation of energy intake in controlled environments, overcoming the issue of misreporting of intake.^4^,^5^ In some cases, the time elapsed until the spontaneous request for the next meal has been used as a surrogate for satiety^1^,^6^; however, this time interval reflects, in part, the hormonal and physiological changes related to preload nutrient oxidation.^7^,^8^ Time is also an important cognitive element influencing intake, with humans typically consuming more of a preload when it is known that there will be no access to other foods for a particular amount of time.^6^ On the other hand, when the intermeal interval (IMI) is fixed, the duration of the IMI may be critical in determining the extent of energy compensation^9^ and be subject to the influence of preload characteristics, such as its physical form (i.e., liquid or solid).^2^,^10^

One question that remains unanswered is which time interval following consumption of a preload is the most appropriate for measuring energy compensation and whether this interval is dependent on preload attributes, such as its physical form.^8^,^11^ Physiologically, 20 min appears to be the minimum interval for the first post-absorptive effects of the preload to influence energy levels,^12^ but this is further influenced by food type and subject characteristics.^9^ Assuming that the rate at which nutrients are delivered from the stomach into the small intestine in healthy subjects is approximately 2–4 kcal/min (8.4–16.8 kJ/min),^13^,^14^ then a period of 60–125 min would be required to empty 250 kcal.^15^ This is corroborated by self-selected IMIs (time until request of a meal) of 1 h and 2 h after carbohydrate-rich and fat-rich preloads (1 MJ, 240 kcal).^16^ It is postulated that short intervals will allow the detection of gastric and orosensory effects, while post-absorptive ileal and colonic effects will require longer intervals.^1^,^9^,^17^

In general, the effect of any preload manipulation will tend to diminish as the IMI extends^9^; however, very few studies have analyzed this effect systematically and they have tended to omit the effects of covariates such as the preload's physical form and energy density. In addition, preload studies have traditionally used a wide variety of IMIs (between 5 min and up to 4 h), with considerable variation in the results (probably associated with the different physiological mechanisms involved during eating).^1^ Many of the studies do not justify the selection of time interval and there appears to be only limited, and inconsistent, empirical data reporting on the differential satiating effects of foods by time.^9^,^18^–^20^

Beyond time and physical form, evidence for a differential effect of gender and BMI on energy compensation has also been controversial.^9^,^11^,^21^–^24^ When reported, gender effects have been associated with differences in hormonal levels^25^ and food-related neural processes.^26^,^27^ On the other hand, evidence suggests that energy intake regulation is impaired in older adults compared with younger individuals and perceptions of hunger and satiety may also be decreased.^28^–^31^

It is known that satiety hormones regulate the IMI in lean subjects^7^,^16^,^32^ but how these processes differ in overweight individuals is still unclear.^8^,^33^

The aim of this systematic review of the literature was to quantify the effects of the time interval (IMI) between preload and next meal on energy compensation levels in adults, when analyzed under laboratory conditions. The ways in which this relationship may be modulated by the food's physical form and by other preload attributes such as weight, energy content, and energy density are also investigated. Data on males and females, as well as lean and overweight/obese subjects, were collected, to compare results across subgroups.

It was hypothesized that changes in the IMI would be associated with changes in energy compensation, so that shorter IMIs would be associated with more precise energy compensation, responding to volume effects (stomach stretch receptors), modulated by the food's physical form and, to some extent, energy load and energy density.^19^,^20^,^34^ Compensation was also expected to increase with increasing energy load (post-ingestive effects) and higher volume (lower energy density). Finally, confirmation was sought regarding the reported differences in energy compensation between men and women and between lean and overweight subjects. Preload nutrient composition was not included in the regression analyses due to the initial complexity of the data, but this will be explored in future analyses.

## Methods

### Search strategy

**Figure 1:**
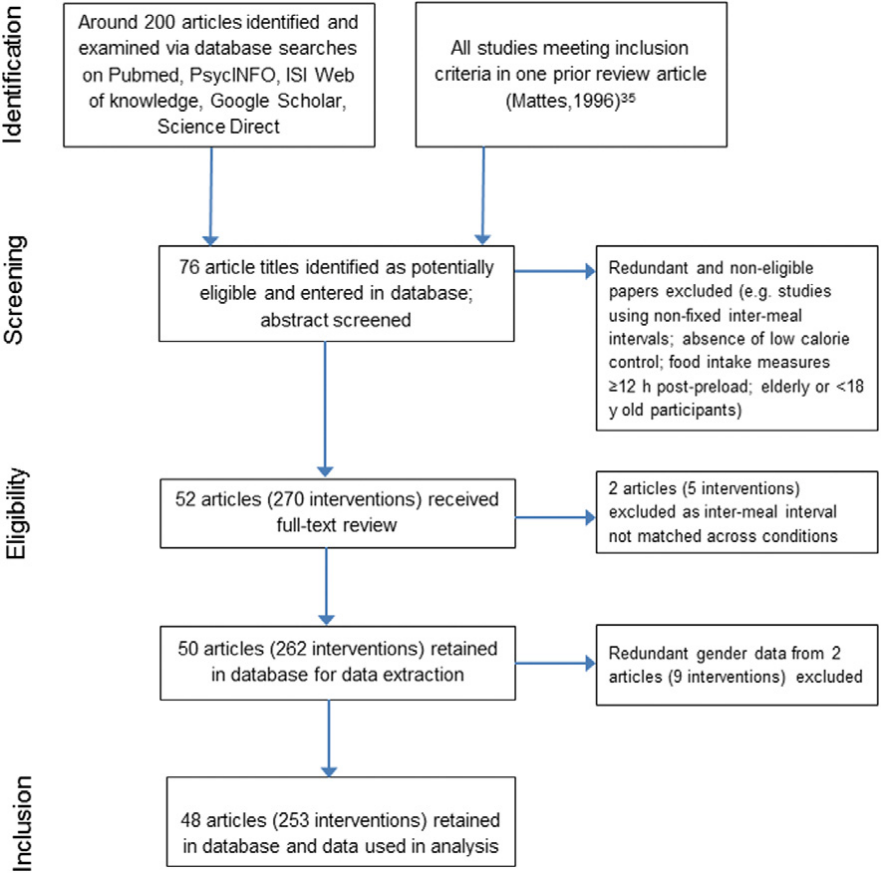
Flow diagram of literature search strategy.

Data from preload studies published in English in paper and electronic format between January 1990 and January 2011 were extracted by four independent investigators (CR, EAR, KG, and NV). It was considered that this time span would allow the identification of sufficient data given the recent popularity of preload designs (e.g., to validate satiety claims for new and reformulated products). Articles were identified by searching the following databases at four different time periods between October 2008 and January 2011: PubMed (US National Library of Medicine National Institutes of Health), PsycINFO (EBSCO Industries, UK), ISI Web of Knowledge (Thomson Reuters, UK), Google Scholar (Google, UK), and Science Direct (Elsevier B.V., UK). Key words used were “energy compensation,” “preload,” “satiation,” “satiety,” “intermeal interval,” “appetite measures,” and combinations of these. The initial searches were complemented with further searches from specific journal websites and by cross-referencing from literature lists in published articles.^35^ About 40% of the identified abstracts were screened by at least two investigators. From these searches, three initial databases were created; these were then merged into one final database in which 100% of the extracted entries were confirmed by at least two investigators (Figure 1).

### Inclusion criteria for publications

Publications were included in the database if they reported oral consumption data for healthy volunteers, ages 18–65 years, with no history of clinically diagnosed eating disorders. Data on percent energy compensation (%EC) versus a no preload or water control or, in its absence, a low-energy control of no more than 150 kcal had to be included. This was done to broaden the inclusion criteria for solid preloads, which were heavily underrepresented in initial searches. The 150 kcal cut-off value was chosen as representative of a small meal.^36^,^37^ Most studies reported energy compensation versus only one control preload. In the few studies where more than one control condition was used, compensation data for only one control comparison were entered into the database to avoid too highly correlated measures of energy compensation.

Studies were included if energy compensation values were provided or if energy intake and preload attributes (i.e., grams, kcal, or kJ) were reported, such that energy compensation could be manually computed. In addition, the IMI had to be reported and be no less than 5 min so the study could be considered a preload design. For the purpose of this review, IMI was defined as the amount of time, in minutes, between the start of the preload and the start of the test meal.^6^ Thus, the time spent by the subjects consuming the preload was included as part of the IMI. Studies with alternative sweeteners or nutrient replacers were included if they met the above inclusion criteria. When appropriate, the alternative sweetener preload condition was used as control preload. Comparisons of alternative sweetener conditions versus water were not included in the review.

Studies conducted in free-living conditions, where subjects spontaneously requested the meals, or in controlled studies, where the IMIs were either not fixed or varied across preload and control foods, were excluded. Studies utilizing nasogastric or other enterally or parenterally infused preloads were also excluded, because orosensory cues were bypassed in these studies, their results may not be comparable to those of studies in which oral cues were present. Studies involving alcohol-containing preloads were also excluded to avoid confounding due to the appetite-stimulating effects of alcohol.^38^

### Data extraction and construction of the database

Mean energy compensation values were extracted from the published source in numerical form from the text or, when available in graphic form only, a numerical value was obtained using an image-to-data tool (http://www.tushar-mehta.com) and subsequently entered into a spreadsheet. Additional information extracted included participants' sex (male, female, or both); BMI group (lean, overweight, obese, or mixed groups); preload weight (g); preload energy (kcal; kJ); preload energy density (kcal/g; kJ/g, including beverages); intermeal time (minutes), and a description of the preload food (physical form, ingredient composition), as well as of the control preload (ingredient and energy content). In regard to physical form, preloads were categorized as liquids (e.g., beverages, broth-type soups, water); semisolids (e.g., yogurts, jelly, fruit puree); solids (e.g., sandwiches, bread, salad vegetables); and composite meals. Composite meals were defined as any semisolid or solid preload that was accompanied by a drink served in a separate container (e.g., sandwich with beverage). Foods composed of solid and liquid parts, such as chunky vegetable soups or chicken and noodle soups, were classed as semisolids.

Whenever not reported, or if reported in another way, %EC was calculated as follows^39^:


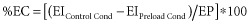


In this equation, EI represents energy intake at the next meal under the control or preload conditions, excluding the energy of the preload itself and of any subsequent meals eaten; EP represents the energy of the preload (or the difference in preload energy when this was >0).

Values of 100% indicate perfect compensation. Values <100% indicate partial compensation, of which values <0% indicate eating additional energy beyond the preload energy content. For example, 50% EC after consumption of a 200 kcal yogurt preload versus water indicates that subjects consumed 100 kcal less at the next meal after eating the preload than after consuming water, while −50% EC indicates that subjects consumed 100 kcal more after the yogurt than after water (referred to as “overeating” in this review). Values above 100% indicate that the preload suppressed subsequent intake to an extent greater than the energy content of the preload (referred to as “overcompensation”). For example, a %EC value of 150% indicates participants consumed 300 kcal less at the next meal after a 200 kcal preload than after the water.

The total weight of the preload included small (50–100 mL) volumes of water when these were administered to diminish aftertaste or reduce mouth dryness, served together or within 5 min of the preload, as it was considered that this amount of water could mix with the preload volume in the stomach. For composite meals, the weight of the beverages (in g) was included in the total weight of the preload and incorporated in the energy density calculation. For instance, in a study^21^ providing preloads that consisted of toast (43 g, 100 kcal) and beverage (591 g, 248 kcal), the energy density of the preload was calculated as total kcal/total g, or 348 kcal/634 g, i.e., 0.55 kcal/g. In studies where subjects were asked to taste and consume a small set of foods before the preload (e.g., to rate pleasantness), energy and weight of the taste test foods were included in the preload energy and weight. In cases where preload weights and energy were not reported (<2% of cases) the weight and energy of the taste foods or any preload component was estimated using the USDA National Nutrient Database release 24 (http://www.nal.usda.gov/fnic/foodcomp/search/).

### Data management and statistics

The Statistical Package for the Social Sciences (SPSS) Version 18 for Windows software (SPSS Inc., Chicago, USA) was used to analyze the data.

The dependent variable was %EC. The predictor variables were as follows: IMI (minutes), preload weight (g); preload energy (kcal); preload energy density (kcal/g); and preload physical form (liquid, semisolid, solid, and composite meal). All variables were treated as continuous data, except for the physical form of the preload, which was treated as a binary dummy variable for each physical form category with liquid as the reference category. BMI was treated as a binary dummy variable to categorize studies with lean subjects only versus studies including lean and overweight/obese subjects. The latter were grouped to allow sufficient sample size for the non-lean studies, which were underrepresented (see Results).

To improve normality, the %EC variable was transformed using the function SQRT (squared root), after collapsing all negative and zero values into zero (as it was considered that a negative %EC and a zero %EC represents a similar behavioral outcome; i.e., the individual either did not respond to the preload energy or actually overate in response to it); the result is referred to hereafter as the energy compensation index (ECI). An ECI value of 10 corresponds to 100% (precise) energy compensation. To illustrate effects across the whole range of data (including negative and null compensation, as well as positive) the untransformed variable, %EC, was used in the descriptive analyses.

Spearman's rank correlation was applied to test the strength and direction of the association between %EC and each of the independent variables (which were also non-normally distributed). The Kruskal-Wallis test was used to compare median %EC values across physical form groups (i.e., liquids, semisolids, solids, and composite meals), and the Mann-Whitney test for differences within physical form category pairs (i.e., median %EC in liquid versus semisolids; liquids versus solids; liquids versus composite meals; semisolids versus solids; semisolids versus composite meals; and solids versus composite meals).

Differences in ECI across physical form groups by gender, BMI category (lean versus other), and IMI category (interventions using IMI of <30 min versus 30–120 min versus >120 min) were explored with ANOVA, including gender and BMI as fixed factors. Zero ECI values (corresponding to null and negative compensation) were excluded from this analysis to achieve homogeneous residual dispersion and, thus, meet the ANOVA assumptions. This exclusion still allows the functional relation between energy compensation and the other variables to be determined when assuming that the preload is effective, thus, representing a means of controlling weight. The Bonferroni correction was applied for post-hoc multiple comparisons. To account for differences in sample size and the presence of repeated measures design across interventions, a weight was applied to each intervention using the following formula: n(1/rep), where “n” is the sample size for the experiment and “rep” is the number of conditions (interventions) each subject was tested on within that experiment, excluding the control condition.^40^

Meta-regression was used to investigate the relative impact of predictor variables on %EC (as ECI) over time in all interventions reporting positive %EC (*n* = 234). Initial visualization of the data indicated that %EC values decreased sharply with the first time intervals; this suggested that log_10_(IMI) would be a more appropriate predictor variable, so this was used in the regression analysis in place of IMI.

The relative impact of log_10_(IMI), preload weight, preload energy, and preload physical form on ECI changes was investigated with and without adjusting for gender and BMI group and also including interaction effects between log_10_(IMI) and physical form. Energy density (ED) is a function of preload energy and preload weight, so ED was investigated in a separate model that did not incorporate weight and energy. This model was also tested twice (with and without adjusting for gender and BMI). All regression analyses were performed by weighted least squares with weights as described for ANOVA.

To further explore the effect of the repeated measures design on the dependent variable (ECI), a sensitivity analysis was conducted by adjusting for the variable indicating the number of repeated measures (range, 1–9) in the regression analyses. The level of statistical significance for all analyses was set at α = 0.05.

### Testing of the models against newly generated data

The predictive capacity of the regression models was tested against data from two interventions reporting positive %EC values for liquids and semisolids as a representation of the most common preload types, published after the original review was completed.^41^,^42^ For this, the published data on IMI, preload weight and energy content, or energy density, and physical form category were entered into each model to calculate the ECI, from which predicted %EC was derived. This was then compared with the %EC values reported for a milk preload^41^ and with the %EC values for a sucrose-sweetened jelly, a sucrose drink, a glucose-fructose drink, and two (one sweet, one acidic) protein-containing jellies and drinks.^42^ It was verified that the reported %EC for all eight preloads was based on the %EC calculation method used in this review, and when this was not the case, it was recalculated and reported in the results. Although the jelly preloads were strictly semisolids, their accompaniment by water led them to be considered “composite meals” to match the criteria used in the review.

## Results

### Database characteristics

The final database contained 253 preload interventions published across 48 independent publications between 1984 and 2011. The preload interventions were 48% liquids^11^,^22^,^24^,^29^,^43^–^68^; 27% semisolid,^9^,^28^,^39^,^43^,^51^–^57^,^69^–^71^ 17% solids or semisolids accompanied by a drink (composite meals),^21^,^47^,^51^,^53^,^56^,^62^,^69^,^72^–^78^ and 8% exclusively solid preloads.^52^,^57^,^64^,^69^,^73^,^79^–^82^ The majority of the studies were in lean subjects^9^,^11^,^21^,^22^,^24^,^28^,^39^,^43^,^44^,^46^,^47^,^49^,^50^,^54^–^57^,^59^,^61^–^63^,^65^–^69^,^71^,^72^,^74^–^82^ (78% versus 22% for non-lean^29^,^45^,^48^,^51^–^53^,^58^,^60^,^64^,^70^,^73^). Studies with males only^9^,^11^,^21^,^22^,^24^,^28^,^44^,^45^,^47^–^49^,^51^,^53^,^54^,^56^,^59^–^63^,^66^–^68^,^70^,^72^,^74^,^76^,^78^,^80^ were overrepresented (48%) compared with studies with only females^9^,^11^,^21^,^22^,^24^,^43^,^50^,^51^,^55^,^56^,^60^,^65^,^66^,^69^,^70^,^81^,^82^ or including both genders^29^,^39^,^46^,^52^,^57^,^58^,^64^,^71^,^73^,^75^,^77^,^79^ (35 and 17% respectively). Only a very small number of interventions^70^ (6%) specifically enrolled restrained subjects, with the majority involving subjects identified as nonrestrained eaters (Table 1).

**Table 1:** Characteristics of studies included in the literature review database

Variable^References^	Preload examples	No. of interventions	Percentage^a^
Physical form			
Liquid preloads^11^,^22^,^24^,^29^,^43^–,^68^	Custom-made beverages (e.g., milkshake with added nutrients), fruit juice, liquid yogurts, milk, soft drinks, smooth soup (e.g., with particles <1 mm), broth, sweetened water	121	48
Semisolid preloads^9^,^28^,^39^,^43^,^51^–^53^,^55^–^57^,^69^–^71^	Yogurts, jelly, pureed food, chunky soups, pasta soups, thickened milkshakes, pate-type food	69	27
Composite meal preloads^21^,^47^,^51^,^53^,^56^,^62^,^69^,^72^–^78^	Any non-liquid served with water or a drink (e.g., cheese, crackers, and fruit juice; meat casserole with glass of water)	43	17
Solid preloads^52^,^57^,^64^,^69^,^73^,^79^–^82^	Vegetables, salad, bread, sandwiches, whole fruit, cheese, meat, pasta	20	8
Sex			
Male participants^9^,^11^,^21^,^22^,^24^,^28^,^44^,^45^,^47^–^49^,^51^,^53^,^54^,^56^,^59^–^63^,^66^–^68^,^70^,^72^,^74^,^76^,^78^,^80^	Data for male participants only	120	48
Female participants^9^,^11^,^21^,^22^,^24^,^43^,^50^,^51^,^55^,^56^,^60^,^65^,^66^,^69^,^70^,^81^,^82^	Data for female participants only	90	35
Both sexes^29^,^39^,^46^,^52^,^57^,^58^,^64^,^71^,^73^,^75^,^77^,^79^	Grouped data for males and females	43	17
BMI and restrained eating			
Lean participants^9^,^11^,^21^,^22^,^24^,^28^,^39^,^43^,^44^,^46^,^47^,^49^,^50^,^54^–^57^,^59^,^61^–^63^,^65^–^69^,^71^,^72^,^74^–^82^	Data for non-overweight, non-obese participants	197	78
Non-lean participants^29^,^45^,^48^,^51^–^53^,^58^,^60^,^64^,^70^,^73^	Includes groups of lean plus overweight or obese subjects	56	22
Restrained eaters^70^	Defined by validated eating behavior questionnaire (10 lean and 5 obese subjects)	15	6
Total interventions		253	100%

a Percentage of number of interventions from total in database.

**Figure 2:**
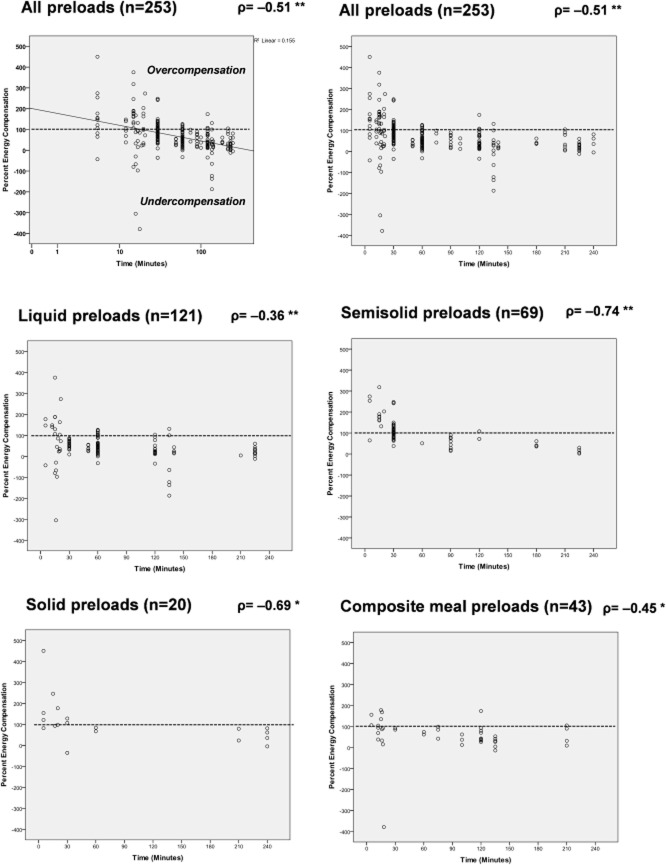
Distribution of %EC values in preload studies (*n* = 253) employing liquid, semisolid, solid, and composite (a solid or semisolid plus a beverage) meal preloads, for intermeal intervals between 5 and 240 min The Spearman's rho coefficient is indicated. *Y*-axis values of 100% indicate perfect compensation (dotted line). Values <100% indicate undercompensation, with values <0% indicating consumption of additional energy beyond the preload energy content (i.e., “overeating”). Values above 100% indicate the preload suppressed subsequent intake to an extent greater than the energy content of the preload (i.e., “overcompensation”). Percent energy compensation is graphed against the Log(Time) on the first graph to improve the fit of the linear regression line. Log(Time) is not used in the other examples to facilitate interpretation of the IMI. * Significant correlation at the *p* < 0.01 level; ** *p* < 0.001.

The median %EC for all 253 foods was 62% (interquartile range [IQR], 74). The %EC values ranged from −379% (overeating, i.e., %EC < 0) to 450% (overcompensation, i.e., %EC > 100). Overeating was restricted predominantly to liquid preloads consumed up to 150 min before the next meal (Figure 2), and was observed in 11% of all liquid preloads (i.e., 13 of 121). The median preload energy, weight, and energy density across all preloads were 263 kcal (IQR, 197), 396 g (IQR, 200), and 0.63 kcal/g (IQR, 0.60), respectively (Table 2).

**Table 2:** Range, median, and interquartile range (IQR) for %EC, IMI, preload weight, preload energy content, and preload energy density of the studies included in the database, overall and by texture subgroup

Preload type	%EC	IMI (min)	Weight (g)	Energy (kcal)	ED (kcal/g)
All foods (*n* = 253)					
Range	−379 to 450	5 to 240	24 to 1,225	37 to 1,175	0.18 to 3.02
Median	62	60	396	263	0.63
IQR	74	90	200	197	0.60
Liquids (*n* = 121)					
Range	−305 to 375	5 to 225	95 to 800	37 to 800	0.18 to 1.66
Median	43^a^	60	415	200	0.45
IQR	64	90	200	150	0.4
Semisolids (*n* = 69)					
Range	2 to 318	5 to 225	100 to 750	39 to 800	0.32 to 1.47
Median	99^b^	30	350	357	0.75
IQR	66	60	150	220	0.56
Composite meals (*n* = 43)					
Range	−379 to 178	5 to 210	54 to 1,225	48 to 1,175	0.21 to 1.91
Median	62	100	410	274	0.63
IQR	60	104	363	176	0.51
Solids (*n* = 20)					
Range	−36 to 450	5 to 240	24 to 693	50 to 658	0.33 to 3.02
Median	83	30	260	307	1.33
IQR	85	195	190	347	1.82

a The median %EC in liquids differs from semisolids with *p* < 0.001, and from solids with *p* < 0.05.

b The median %EC in semisolids differs from composite meals with *p* < 0.01.

When grouped by physical form, the median %EC values were as follows: 99% for semisolids (IQR, 66); 83% for solids (IQR, 85); 62% for composite meals (IQR, 60); and 43% for liquids (IQR, 64). These differences were significant when tested with the Kruskal Wallis test (X^2^ = 38.07; *p* < 0.001). Significant differences were detected between %EC medians in liquids versus semisolids and solids; and in semisolids versus composite meals (Table 2). Liquids had the lowest energy load, highest volume, and lowest ED, while solids had the highest ED, as expected. The median IMI was 60 min for all food interventions (IQR, 90 min). IMI ranged widely within all physical form categories (Table 2).

### Association between energy compensation and IMI and preload physical form

Percent energy compensation correlated significantly with IMI for all food interventions (*n* = 253, *p* < 0.001). The association was strongest for semisolid foods but was robust across physical form subgroups (Figure 2).

For the studies reporting positive %EC only, the ECI correlated well with the IMI (*r* = −0.430, *p* < 0.001, *n* = 234) and even better with log_10_(IMI) (*r* = −0.475, *p* < 0.001), indicating that log_10_(IMI) was probably a better predictor variable than IMI. Time interval alone as log_10_(IMI) explained 23% of the variance in ECI in a simple regression model. When explored by gender and BMI subgroups, ECI correlated significantly with log_10_(IMI) in females (*r* = –0.513, *p* < 0.001), males (*r* = –0.499, *p* < 0.001), lean subjects (*r* = –0.395, *p* < 0.001), and in the non-lean group (*r* = –0.731, *p* < 0.001). These models, however, do not take into account possible modulating effects of other variables such as physical form.

Differences in ECI across physical form subgroups were significant for the whole sample (*F*_3_ = 14.66, *p* < 0.001). Post-hoc subgroup comparisons revealed that ECI for liquids (mean ± SEM 7.12 ± 0.27) was significantly lower than for semisolids (9.52 ± 0.41, *p* < 0.001) and solids (10.86 ± 0.64, *p* < 0.001); but not different from composite meals (7.81 ± 0.47, *p* > 0.05); while composite meals differed significantly from semisolids (*p* < 0.05) and solids (*p* < 0.01). There was no interaction between physical form and gender (*p* = 0.08) or physical form and BMI (*p* = 0.30).

**Figure 3:**
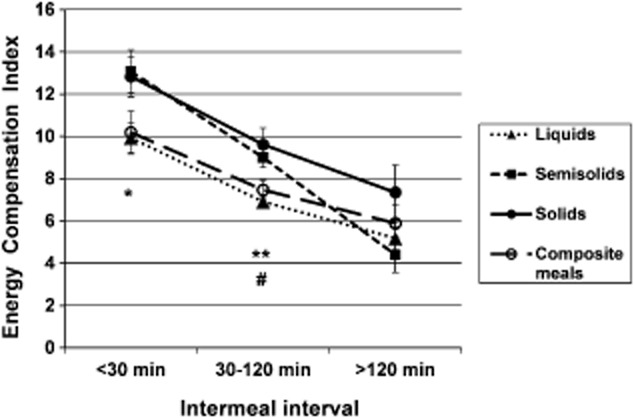
Mean (SEM) energy compensation index in studies using IMIs of up to 30 min (*n* = 50), 30–120 min (*n* = 145), and >120 min (*n* = 39) by preload physical form category Only studies reporting positive energy compensation (EC) are included. An ECI value of 10 corresponds to 100% (precise) energy compensation. ECI values >10 indicate overcompensation. * Liquid preloads differ from semisolid preloads with *p* < 0.05; ** liquid preloads differ from semisolid preloads with *p* < 0.001 and from solid preloads with *p* < 0.01; ^#^ trend for semisolid preloads to differ from composite meals with *p* = 0.077.

IMI was divided in three meaningful categories (with cut-offs at 30 and 120 min representing relatively short and long IMIs commonly used in the literature) and found to have a significant interaction with preload physical form (*p* = 0.014). Hence, a subgroup analysis by categories of IMI was conducted by ANOVA, which revealed a significant main effect of physical form within interventions using IMIs < 30 min (F_3_ = 3.92, *p* < 0.05) and IMIs 30–120 min (F_3_ = 8.97, *p* < 0.001) but not in studies with longer IMIs (>120 min). Post-hoc tests revealed differences in liquids versus semisolids for studies using IMIs < 30 min, which were maintained when IMI extended to 30–120 min. For this time interval, liquid preloads differed also from solids. Beyond 120 min, there were no differences across physical forms (Figure 3).

### Correlation between energy compensation and preload weight, energy content, and energy density

**Figure 4:**
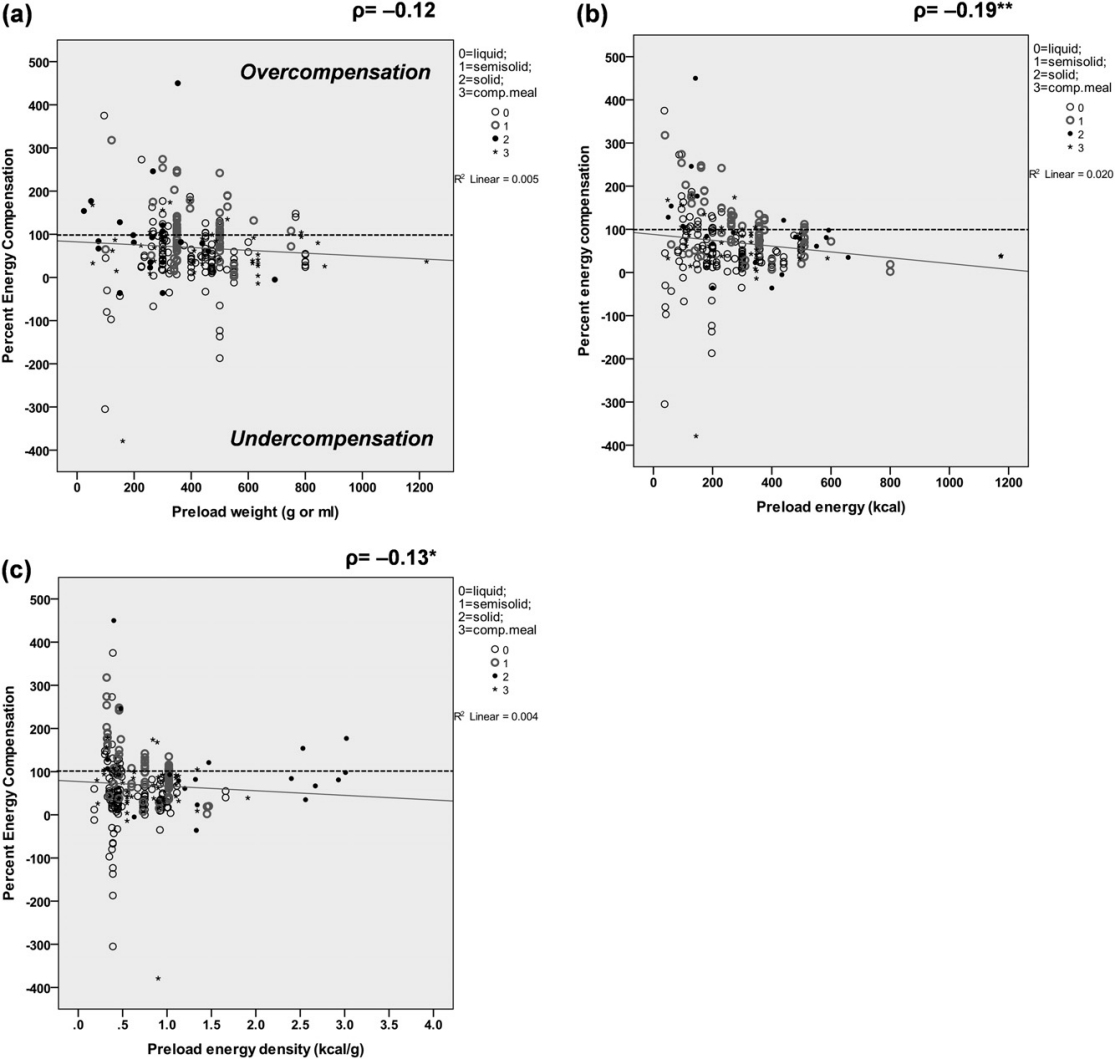
Distribution of %EC values in preload studies (*n* = 253) according to preload weight, preload energy content, and preload energy density The Spearman's rho coefficient is indicated. *Y*-axis values are to be interpreted as for Figure 2, in reference to the 100%EC line (dotted line). * Significant correlation at the *p* < 0.05 level; ** *p* < 0.01.

No significant correlations were detected between %EC and preload weight for the whole dataset or for physical form subgroups (Figure 4a).

A significant correlation was identified between %EC and preload energy content for all food interventions (*p* < 0.01) (Figure 4b). This correlation was strongest for semisolid foods (rho = –0.549, *p* < 0.001) and was still significant for solid foods (rho = –0.541) and composite meal preloads (rho = –0.331; both *p* < 0.05) but not liquid preloads (*p* > 0.05).

A significant correlation was identified for %EC with preload energy density for all food interventions (*p* < 0.05), and this was driven mainly by the semisolid preloads (rho = –0.544; *p* < 0.001) (Figure 4c). The remaining correlations were non-significant (liquids; composite meals and solid preloads all *p* > 0.05).

After transformation, ECI still correlated significantly with preload energy content (r = –0.166; *p* < 0.01), but not with preload weight or energy density.

### Relative contributions of time and preload characteristics to changes in percent energy compensation

Meta-regression analysis was used to investigate the relative impact of IMI and preload characteristics on the ECI variable (*n* = 234). Both models used liquid as the reference physical form. In the first model, ECI was regressed on log_10_(IMI), preload weight and energy content and in the second one, on log_10_(IMI) and energy density. Adjusting for BMI and gender did not significantly affect the results; therefore, these two covariates were dropped in subsequent analyses. Interactions between log_10_(IMI) and physical form were also included in the model initially but appeared to be non-significant and were subsequently dropped. The results of each model analysis are shown in Table 3. In the first model, the variables with the largest effect in decreasing order of standardized coefficient's size, were log(IMI), semisolid physical form, solid form, energy content, preload weight, and, lastly, composite meal form. All the variables made a significant contribution to the model (*p* < 0.05), which explained 50% of the variance in ECI (*R*^2^ *=* 0.499, *F*_6_ = 37.67, *p* < 0.001) (Table 3). The effect of time should be interpreted in a multiplicative way in that the same effect of about −0.5 log_10_(IMI) on ECI would occur between 1 and 10 min,10 and 100 min, and 100 and 1,000 min.

**Table 3:** Linear regression coefficients (B) and standardized coefficients (β) for the association between ECI and preload study variables across a sample of 234 food interventions (weighed by the weighted least squares method)

Variable^a^	B	SE for B	95% confidence interval for B	Standardized β coefficients	*P* value
Model 1^b^					
(Constant)	14.462	0.737	(13.009–15.914)		<0.001
LogIMI (min)	−4.207	0.424	(−5.042–−3.371)	−0.514	<0.001
Weight (g or mL)	0.002	0.001	(0.000–0.005)	0.139	0.017
Energy (kcal)	−0.004	0.001	(−0.007–−0.002)	−0.235	<0.001
Semisolid = 1 (Other = 0)	2.284	0.389	(1.518–3.050)	0.296	<0.001
Solid = 1 (Other = 0)	3.198	0.585	(2.046–4.350)	0.287	<0.001
Comp. meal = 1 (Other = 0)	1.016	0.428	(0.173–1.860)	0.120	0.018
Model 2^c^					
(Constant)	15.466	0.711	(14.065–16.868)		<0.001
LogIMI (min)	−4.285	0.399	(−5.071–−3.499)	−0.523	<0.001
Energy density (kcal/g)	−1.548	0.341	(−2.220–−0.877)	−0.257	<0.001
Semisolid = 1 (Other = 0)	2.134	0.382	(1.382–2.886)	0.276	<0.001
Solid = 1 (Other = 0)	4.283	0.653	(2.998–5.569)	0.384	<0.001
Comp. meal = 1 (Other = 0)	1.105	0.419	(0.280–1.930)	0.130	0.009

a The liquid form was used as the reference physical form in each model.

b ECI = 14.462 − (4.207*logIMI) + (0.002*g) − (0.004*kcal) + (2.284 if semisolid) + (3.198 if solid) + (1.016 if composite meal).

c ECI = 15.466 − (4.285*logIMI) − (1.548*kcal/g) + (2.134 if semisolid) + (4.283 if solid) + (1.105 if composite meal).

*Abbreviations*: SE, standard error.

Results for the model including preload ED were similar, with log(IMI) being the strongest contributing variable, followed by solid physical form, semisolid form, and then ED. Overall, this model also explained 50% of the variance in ECI (*R^2^* *=* 0.502, *F*_5_ = 45.94, *p* < 0.001) (Table 3).

Adjusting for the variable indicating the number of repeated measures in the regression analyses had virtually no change on the direction or magnitude of the coefficients and the *p*-values (only a small increase in the ECI of 0.195 per repeated measure (*p* = 0.046) was detected in the model with energy and weight, with no significant effects detected for the model with ED).

### Predictive capacity of the models

Based on the two models generated in this review, the predicted %EC for the preloads examined by Maersk et al.^41^ and by Akhavan et al.^42^ were within the 95% confidence interval for the reported %EC; the two exceptions were the acid protein jelly and the acid protein drink preloads, for which the predicted values were slightly below the lower end of the reported %EC confidence interval (Table 4).

**Table 4:** Comparison of reported against predicted %EC in eight interventions not included in the review

Reference	No. of subjects	Intervention and control preloads^a^	IMI (min)	Reported mean %EC ± s.e.m.	Reported 95%CI for mean	Predicted %EC (based on ECI)
Maersk et al. (2012)^41^	24 obese subjects	500 mL (950 kJ, 227 kcal) of semi-skimmed milk against 500 mL water	240	14.7% ± 29.0%	(−42.1, 71.5)	21% (model 1) 21% (model 2)
Akhavan et al. (2011)^42^	15 lean men	300 mL (1,340 kJ, 320.1 kcal) of sucrose-sweetened jelly against 300 mL of sucralose-sweetened water (<0.5 kcal)	60	32.2 ± 19.2% (30.2%)^b^	(−5.4, 69.8)	57% (model 1) 59% (model 2)
		300 mL (1,340 kJ, 320.1 kcal) of sucrose drink against same control	60	35.3 ± 21.6% (33.1%)^b^	(−7.0, 77.6)	42% (model 1) 44% (model 2)
		300 mL (1,340 kJ, 320.1 kcal) of glucose-fructose drink against same control	60	35.8 ± 23.7% (33.6%)^b^	(−10.7, 82.3)	42% (model 1) 44% (model 2)
Akhavan et al. (2011)^42^	14 lean men	300 mL (1,255 kJ, 300 kcal) of sweet whey jelly against 300 mL water	60	82.1 ± 16.6%	(49.6, 114.6)	58% (model 1) 61% (model 2)
		300 mL (1,255 kJ, 300 kcal) of acid whey jelly against 300 mL water	60	112.8 ± 19.9%	(73.8, 151.8)	58% (model 1) 61% (model 2)
		300 mL (1,255 kJ, 300 kcal) of sweet whey drink against 300 mL water	60	78.7 ± 22.0%	(35.6, 121.8)	43% (model 1) 45% (model 2)
		300 mL (1,255 kJ, 300 kcal) of acid whey drink against 300 mL water	60	89.2 ± 19.9%	(50.2, 128.2)	43% (model 1) 45% (model 2)

a The sugar-sweetened beverage preload in Maersk et al. was not used as it reports an average EC below zero (overeating). The preloads in Akhavan et al. were served followed by 100 mL of water, which was added to the weight to calculate predicted %EC.

b %EC was calculated following this review's methodology (incorporates full weight and EC for both intervention and control preloads).

*Abbreviations*: ECI, energy compensation index; s.e.m., standard error of the mean.

## Discussion

The findings of this review demonstrate that under controlled laboratory conditions, the IMI is a strong determinant of energy compensation differences. Moreover, when examined in the context of BMI, gender, preload physical form, and preload weight, energy, and energy density, the IMI is the strongest contributing variable, followed by the physical form of the preload and its energy density. The regression models generated are able to explain up to 50% of the variance in %EC. Nearly half of the interventions consisted of liquid preloads, while solid preloads were underrepresented (<10%). The majority of studies employed lean individuals (frequently men), while less than 25% of interventions involved overweight and/or obese participants. In agreement with this review's main hypothesis, energy compensation decreased in all types of preloads the longer the IMI. Compensatory behavior appeared to decrease faster over time with semisolids and solids than with liquids, but results for solid preloads need to be interpreted carefully due to the small number of interventions identified. Liquid preloads were associated with incomplete energy compensation (%EC < 100) more frequently than other preloads, as documented previously.^10^,^19^,^21^ Of the 253 interventions examined, 144 (57%) reported incomplete compensation (i.e., energy compensation levels of 75% or less), and these were mainly associated with liquid preload results. Thus, among the liquid preload interventions, 73% (89 of 121) reported incomplete compensation. In contrast, 58% of the composite meal interventions, 35% of the solid, and 33% of the semisolid reported incomplete compensation of 75% or less. This suggests that studies with semisolid or solid preloads are more likely to result in close to, or precise, compensation (i.e., 76–100%) than studies including liquids alone, or as part of a meal. Further, overeating (%EC < 0) was detected in 11% of the liquid preloads (13 of 121 interventions) but in only 2 of the 43 composite meal interventions and in none of the semisolid interventions. Although the frequency of overeating with solid preloads was high (15%; 3 of 20), the sample of solid interventions may have been underrepresented. Overcompensation (%EC > 100), however, occurred in all preload types when the IMI was very short (<30 min).

### Role of preload physical form on energy compensation

Overall, the results confirm the reported weak satiating effects of liquid preloads and their effects on energy compensation.^2^,^10^,^19^,^83^–^86^ Semisolids incorporate two properties that have been associated with satiety and that are not present in combination in any other physical form; these are the relatively large water content and the textural effects associated with chewing and affecting eating rate.^83^,^85^ These properties may explain the enhanced satiating capacity of semisolids over other preloads at short time intervals. In contrast, beverages have a high water content, but are consumed faster than semisolids,^85^,^87^ while solids are associated with greater chewing effort and longer oral exposure,^88^ which has been linked with higher satiety than liquids.^62^,^82^,^89^ The effects of eating rate and oral exposure relate, in part, to the concept of the taste system as a nutrient system (i.e., the sensory properties of food having an effect on satiety), as recently demonstrated in controlled studies.^90^,^91^ Sensory but also cognitive aspects (i.e., odor, visual texture, food size, and the perception of food as something to quench thirst versus hunger) have been implicated in different oral, hormonal, and gastrointestinal responses affecting the satiating properties of liquids, semisolids, and solids.^6^,^85^–^88^,^92^,^93^ It is, therefore, important to be cautious when categorizing the product's physical form, especially for foods at the interface of categories (e.g., yogurts, soups, and custards).

### Analyses with studies reporting positive energy compensation

Results from the regression analyses support the hypothesis that the IMI is a major explanatory variable for changes in ECI, while the relationship between the log of time and ECI is shifted but not modified by physical form (based on the significant main effect of physical form and non-significant interactions between time and physical form). Accordingly, in these models, the regression lines for different types of physical forms are parallel; however, as the time variable has been transformed to a logarithmic scale to allow better fit of a linear model, the actual relationship with time is non-linear. This result is consistent with findings from free-living studies, which show a positive correlation between IMIs and the energy content of the meal.^37^ As the contribution to changes in %EC was significant for both time and physical form, and together they contribute to a large amount of the variability in %EC, it can be concluded that both factors are important to consider when designing laboratory studies on energy compensation. For example, while IMIs shorter than 30 min appear to produce large variability in individual compensatory responses, especially for liquid preloads, the effects of physical form and energy dissipate beyond 2 h when the energy content of the stomach has decreased and hunger signals become stronger.^37^ Thus, studies employing shorter or longer time gaps may not allow the detection of significant responses to texture manipulations or physical form contrasts due to excess variability or small size effects. To avoid such limitations, a possible useful starting point when designing a preload study would be to consider intervals of 30–120 min as potentially maximizing energy compensation. Within this timeframe, the most appropriate interval will depend on the study hypothesis, combined with specific preload characteristics (i.e., if physical form can be chosen, semisolid or solid should be considered first to maximize compensation). Beyond the variables studied in this review, the potential role of other factors should also be considered, such as the preload macronutrient composition, energy needs of the subjects, and nature (i.e., restricted meal versus buffet) of the test meal.^1^,^48^,^94^

The effects of physical form varied across time intervals (i.e., from <30 min to >120 min), which suggests that such physical effects are subject to the effects of time and confirming that time is a main factor (Figure 3). Thus, in the first 30 min after ingestion, semisolids and solids are the physical forms of food that are able to elicit the most precise compensation; however, semisolids quickly become less satiating beyond this time, and by 2 h, all foods elicit similar levels of incomplete compensation.

Preload ED was a weaker predictor of changes in %EC than time and physical form, but its contribution was still significant. From model 1, described above, it appears that the contribution of ED toward energy compensation may depend more on the energy content of the preloads and slightly less on the volume. A significant correlation was found between both %EC and ECI with preload energy, but not with preload weight. However, contrary to our expectations, and to the well-known hypothesis that mass/volume is a more important determinant of energy intake than energy content,^95^ the higher energy preloads were associated with less, not more, compensation. Some of the preload studies analyzed did not match the preload foods for palatability. As palatability correlates positively with energy density,^89^ and has been associated with increased consumption through sensory stimulation,^96^,^97^ interactions between palatability and energy density may have occurred, which could have confounded the results. In addition, the significant correlation between energy content and energy density present in our dataset (rho = 0.71, *p* < 0.01) may partly explain these results. Energy density incorporates both energy content and weight/volume simultaneously, either of which can influence %EC to a different extent. Employing a combination variable instead of weight and energy may have eliminated part of the confounding.

It is important to note two traits of the prediction models described here. First, the direction of the change in %EC (as ECI) is as predicted. That is, the longer the IMI, and the higher the ED, the smaller the %EC observed. This last result is probably associated with volume effects being stronger shortly after eating,^98^ particularly for liquid and semisolid foods, which, at a lower ED, would elicit stronger stomach stretch receptor signals than solids,^99^ in addition to different cognitive and sensory processes.^86^ Second, the magnitude of the predicted change in %EC is similar whether the model includes weight and energy content or ED. These two facts suggest that these models, albeit with a degree of error, appear robust in estimating changes in %EC. To confirm this, the models were tested against newly generated data from two recent publications, which were not included in the review; in seven of the eight interventions examined, the predicted value fell within the 95%CI of the reported value (differences in the jelly preload disappeared when it was categorized as semisolid).

Although both models can, in principle, be applied to predict changes in energy compensation under laboratory conditions for hypothetical preloads, in practical terms, model 1 is more specific because it defines both weight and energy content. For instance, a dairy product preload served 90 min before a test meal, weighing 150 g, with an energy content of 150 kcal, would be expected to elicit, on average, between 60% and 70% compensation if consumed as a semisolid (e.g., fromage frais or yogurt) but only 30–35% compensation if provided as a liquid yogurt beverage. Similarly, a 28 g (132 kcal) granola bar consumed with 200 mL water, would be expected to elicit precise compensation up to 20 min, based on model 1. These predictions have an associated error that depends on the level of unexplained dispersion (1- *R^2^*), i.e., variability in responses to preload interventions that is not explained by the linear regressions.

No clear effects of BMI category or sex were observed using this dataset. While %EC appeared to decrease faster in the non-lean group than in the lean group when using the whole dataset, such effects disappeared when analyzed in the context of other parameters (e.g., physical form, time). Some studies used preloads that were adjusted to the subject-specific energy requirements (including gender and BMI), while others did not. It is possible that these methodological differences, plus the inclusion of subjects with a wide range of BMIs in the non-lean category (as opposed to only overweight or obese, underrepresented in our dataset) could have masked some of the effects of BMI. A larger sample size for studies with exclusively overweight and obese subjects may overcome this problem.

### Model validity and study limitations

The meta-regression analysis in this review has some important limitations. First, it was based on study-level summary results (i.e., average %EC) that did not include any information on the variability of the study-level estimates of energy compensation, which would have allowed fixed and random effects meta-analyses to be applied.^100^ Moreover, some of the summary-level results were obtained on the same group of subjects (with different preloads); therefore, some of the energy compensation summary values are likely to be correlated. These two issues were addressed by weighing the studies so that the weight of a study in the multiple regression analysis is directly proportional to sample size and inversely proportional to the number of times a study was repeated on the same subjects. It is recognized that the influence of a study estimate on the results of a meta-regression analysis depends on its standard errors and that, ideally, meta-regression analysis of such a dataset would be performed on individual participants' data to account for the within-subject variability; however, data on individual variability was not directly available for most of the preload interventions included in the analysis. The lack of such data also prevented us from taking into account the dependence of some of the effect size estimates in the meta-regression. These limitations of the model, which were partially addressed by the weighting, might have caused a decrease in the standard errors of the final estimates but would be unlikely to modify the direction and interpretation of the relationships between energy compensation, IMI, and preload physical form. The sensitivity analysis further confirmed that a repeated measures design had a very minimal impact on the results. Nonetheless, because there was a small significant increase in the ECI as the repeated sessions increased, this is probably an important variable to consider when planning future studies (i.e., to minimize learning effects that may lead subjects to compensate more as the number of exposures increases).

The liquid and semisolid preloads were overrepresented, which means the models are less accurate for solids and composite meals. Also, the majority of the studies included were in lean, adult subjects <65 years, making extrapolation of findings to the obese population and to other age groups less accurate. Only a very small number of interventions specifically enrolled restrained subjects, with the majority involving non-restrained (and presumably not disinhibited) eaters, such that these findings are not generalizable to individuals displaying dietary restriction or disinhibition. Finally, the models are able to explain only 50% of the variability in energy compensation for studies employing time gaps of up to 4 h and using a fixed IMI rather than spontaneous IMI. More research is needed to investigate what other factors are associated with changes in compensation, and in which direction these occur, as well as how these vary over the course of the day, or for longer periods of time as a reflection of free-living conditions. While these will offer complexity that needs to be addressed through future analyses, they are nonetheless relevant, with examples including the following: the macronutrient composition of the preload, including the protein and fiber content and glycemic properties; the preload palatability; whether the preload energy content was fixed versus proportional to the energy needs of the subject; and the format of the subsequent meal, i.e., buffet-style or restricted item.

## Conclusion

Overall, the results of this study show that energy compensation under laboratory conditions is affected by a combination of time factors and the physical form of the preload. In studies employing the preload paradigm with semisolid, solid, and composite meal preloads, and in some cases liquid preloads, participants were able to compensate all or part of the energy consumed in the preload, or even overcompensate, when the time interval to the next meal was short (<30 min). However, this compensatory behavior became weaker with longer IMIs and less sharp, depending on the preload's physical form. On average, compensation drops below 60%, irrespective of physical form, when the IMI exceeds 2 h. To our knowledge, these are the first empirical data demonstrating a significant, robust effect of time over other preload attributes in energy compensation studies. Using the models described here, it was possible to quantify the relative impact of various factors on energy compensation when present simultaneously in the same study design; this provides new information beyond the effects of the individual variables on their own. Based on a large number of studies, it can be concluded that the preload paradigm is a useful methodology to assess the effects of particular food attributes on energy compensation in the short term and under controlled environmental conditions. These results may assist in the design and interpretation of experimental studies investigating the relative efficacy of new interventions to enhance satiety, such as functional foods or commercial weight-management products.
